# Effect of Cr Element in Gas-Shielded Solid Wire for Oil and Gas Long-Distance Pipeline on Microstructure and Low Temperature Toughness of Weld

**DOI:** 10.3390/ma17194704

**Published:** 2024-09-25

**Authors:** Rui Hong, Hai-chun Liu, Xiao-dan Zhu, Neng-sheng Liu, Shu-biao Yin, Qi-lin Ma, Shu-jun Jia

**Affiliations:** 1 Metallurgy and Energy Engineering, Kunming University of Science and Technology, Kunming 650000, China; 15105633440@163.com (R.H.);; 2 Department of Structural Steels, Central Iron and Steel Research Institute, Beijing 100081, China; 3 PipeChina, Ltd., Beijing 100101, China; 4 China Petroleum Pipeline Engineering Corporation, Langfang 065000, China

**Keywords:** gas-shielded solid wire, oil and gas long-distance pipeline, Cr element, toughness, acicular ferrite

## Abstract

In this paper, the influence of Cr element on the mechanical properties of welded joints of gas-shielded solid wire used in oil and gas long-distance pipelines was studied by means of tensile test, impact test, and hardness test, and the microstructure and crack propagation path of weld were characterized by means of an optical microscope, scanning electron microscope, and electron backscattering diffraction. The results show that with the addition of Cr, the strength and toughness of the weld are significantly improved, in which the tensile strength is increased from 607 MPa to 656 MPa, and the impact toughness is increased from 126.37 J to 223.79 J. The proportion of the ferrite side plate in the weld structure is reduced by about 20%, and the effective grain size of acicular ferrite is reduced by about 15%. The reason is that the addition of the Cr element improves the hardenability of the weld structure, inhibits the formation of the ferrite side plate, and promotes the effective refinement of acicular ferrite, which increases the proportion of high-angle grain boundaries in the weld, effectively hindering the crack propagation, improves the crack propagation work, and thus improves the strength and toughness of the weld.

## 1. Introduction

With the rapid increase in demand for oil and natural gas in society, the construction of long-distance pipelines has begun to develop towards large-diameter, long-distance, high-pressure, and high-steel-grade directions [1,2,3]. However, oil and gas resources are often distributed in high dimensional cold regions, with winter environmental temperatures typically below −10 °C, posing a serious challenge to the quality of welds, especially low-temperature toughness [4,5,6,7,8]. To address this issue, advanced welding techniques and materials are needed to ensure the stability and reliability of welds in low-temperature environments.

Fully automatic welding has advantages such as speed and efficiency, stable quality, easy operation, beautiful appearance of weld seam, and stable internal quality of weld seam mechanical properties. It has become an important means for the future development of digital pipelines and smart pipelines [9]. At present, fully automatic pipeline welding technology has gradually been widely applied in oil and gas long-distance pipeline engineering [10]. Welding materials are the “needle and thread” of engineering and equipment manufacturing, playing a crucial role in major projects and equipment [11]. In fully automatic welding technology, welding materials are also one of the important elements that affect the performance of the weld seam. Among them, gas-shielded solid welding wire is the most important welding material, which often determines the performance of the weld seam. In order to meet the higher-level demand for welding materials in the construction of oil and gas long-distance pipelines, it is necessary to continue technological innovation, research, and development in the field of gas-shielded solid welding wires, and continuously improve the performance and quality of products.

With the construction and development of oil and gas long-distance pipelines, research on the welding field of pipeline steel is also constantly deepening. Most of the existing research on gas-shielded solid welding wires is focused on welding and production processes [12,13,14]. For example, Lan Jiuxiang et al. conducted systematic experiments and analysis on the melting electrode gas-shielded welding process of 1000 MP high-strength steel solid welding wires and deeply explored the effects of welding current and preheating temperature on the microstructure, hardness distribution, and mechanical properties of joints [13]. Wang et al. studied the effect of different production processes on the performance of gas-shielded solid welding wires. The study showed that as the wire diameter decreased and the spinning temperature decreased, the strength gradually increased, and the ductility decreased [14]. Few scholars have studied the influence of its chemical composition. However, the influence of different alloy elements on the microstructure and mechanical properties of welds in other welding fields has been fully evaluated [15,16,17,18,19,20,21,22,23,24,25,26]. Research has also been conducted on the influence of Cr elements on the microstructure and properties of weld metal. Cr element is a carbide-forming element and a commonly used strengthening element in steel, which can improve the hardenability of welds and enhance the tensile strength of weld joints. But it is unfavorable for toughness and plasticity. Hee jin Lee et al. studied the effect of Cr content on the formation of acicular ferrite in low-carbon steel welds by submerged arc welding. They found that samples with higher chromium content had lower content of Weinstein ferrite, while the proportion of acicular ferrite increased, and acicular ferrite improved the toughness of the weld [27]. Wang Feng et al. explored the main influencing factors of the unstable impact performance of semi-automatic welding seams with self-protection flux-cored welding wires from the perspective of alloy elements. Research has shown that Cr can significantly improve the quenching tendency of welds and delay the pearlite transformation, making it easier for the weld metal to obtain a bainitic structure [28]. Cui Li et al. investigated the effect of trace element Cr on the microstructure, mechanical properties, and corrosion performance of corrosion-resistant steel welded joints. The results showed that the trace addition of Cr element to a certain extent suppressed pre-eutectoid ferrite and refined needle-shaped ferrite. The tensile strength of welds was higher than that of the base material, but the impact level did not change significantly, and the hardness distribution was similar [29]. Cai Yangchuan et al. found that when w (Cr) > 1.2%, with the increase in Cr content, the strength of the weld metal gradually increases, while the toughness gradually decreases. When w (Cr) = 1.2% in the deposited metal of the weld, a large amount of fine and uniform granular M/A structure can be obtained, thereby improving the strength and toughness of the weld [30]. However, the specific effect of Cr element in gas-shielded solid wire used in oil and gas long-distance pipelines on weld properties is still insufficient, and the Cr content in previous studies is basically higher than 0.5%wt. In this paper, under the premise of low Cr content (w(Cr) < 0.3%), the influence of Cr element in gas-held solid welding wire used for oil and gas long-distance pipelines on weld metal microstructure properties is analyzed. The energy consumed at different stages of crack propagation and the influence of microstructure on the crack propagation path were studied systematically and intuitively, and the microstructure and crack propagation were connected. The purpose is to improve the strength of the weld in a more economical way while maintaining good low-temperature toughness of the weld and to provide help for the composition design of gas–solid welding wire materials for oil and gas long-distance pipelines.

## 2. Experimental Materials and Methods

### 2.1. Welding Material

As filler materials, two types of independently designed and manufactured gas-shielded solid wires are utilized. Table 1 shows the chemical composition of the welding wire. With the multi-layer, multi-pass gas-shielded welding method, the weld metal is obtained. Before welding, surface welding is performed on both sides of the weld metal to prevent the base metal from becoming diluted. Table 2 illustrates the chemical composition of weld metal. The weld joints with 0.25% Cr element added and without Cr element were named as different specimens 1# and 2#, respectively. By comparison with Table 1 and Table 2, it can be found that the content of C in the weld metal has increased. Because the protective gas used contains CO_2_, the oxidation of the protective atmosphere during arc combustion is strong, so the deoxidizing alloy elements Mn, Si, and Ti in the welding wire have a certain burn loss, and the content of these elements in the weld has decreased.

### 2.2. Instrument Charpy Impact Tests

**Figure 1:**
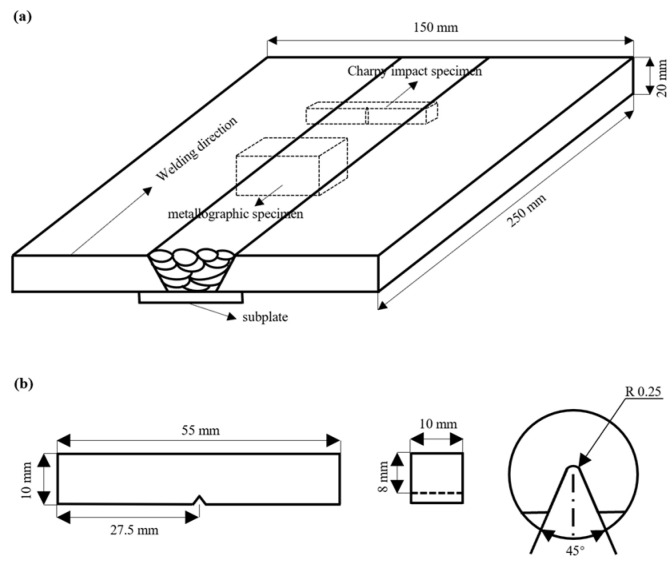
Welding and sampling schematic (**a**) schematic illustration (**b**) dimension.

A size of 55 mm × 10 mm × 10 mm instrument Charpy impact specimen was prepared, and a V-shaped notch with a depth of 2 mm, an angle of 45°, and a radius of curvature of 0.25 mm was processed. The schematic diagram of the sample is shown in Figure 1. The instrument Charpy impact test was carried out according to the standard GB/T 19748-2019 [31].

### 2.3. Microstructure Characterization of Weld and Fracture

Metallographic samples with a size of 30 mm × 20 mm × 20 mm were taken from the welded joint, as shown in Figure 1a. After grinding, polishing, and corrosion with 3% nitrate ethanol solution for about 15 s, FEI Quanta 650FEG (Fremont, CA, USA) thermal field emission scanning electron microscope was used to analyze the microstructure and impact fracture in the test wires. Ten points were selected at different positions in the heart and the FM300 micro-Vickers hardness machine was used to test the hardness (Philadelphia, PA, USA). The load was set to 1 kg and the loading time was 10 s. A maximum value and a minimum value were removed, and the average value was taken to make the microhardness result. The core of the weld metallographic specimen was electrolytically polished with 10% perchloric acid alcohol solution, and the impact fracture of the weld was plated with nickel and then subjected to vibration polishing. EBSD analysis was performed using a field emission scanning electron microscope equipped with an Oxford F-plus backscattering diffractometer with a scanning step of 0.2 μm. The scanning area is 100 μm × 100 μm.

## 3. Experimental Results and Analysis

### 3.1. Weld Metal Structure

**Figure 2:**
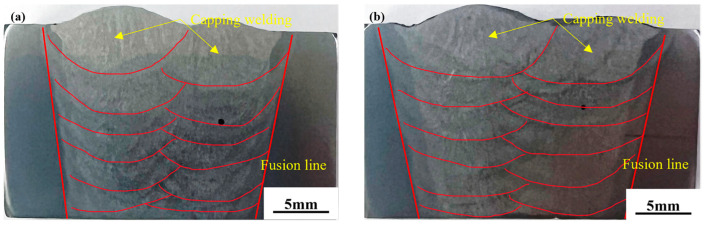
Macroscopic characteristics of weld metal with different chromium contents: (**a**) 1# (**b**) 2#.

The macroscopic morphological features of the two types of weld metals are displayed in Figure 2. There are no discernible differences in the macroscopic morphology of the two types of weld metals, which are produced using the same welding process and twelve welding passes. The welded junction is made up of a columnar crystal zone and a reheat zone based on its macroscopic structure. The white stripes generated during the secondary heating process separate a clear reheat zone from the columnar grain zone.

**Figure 3:**
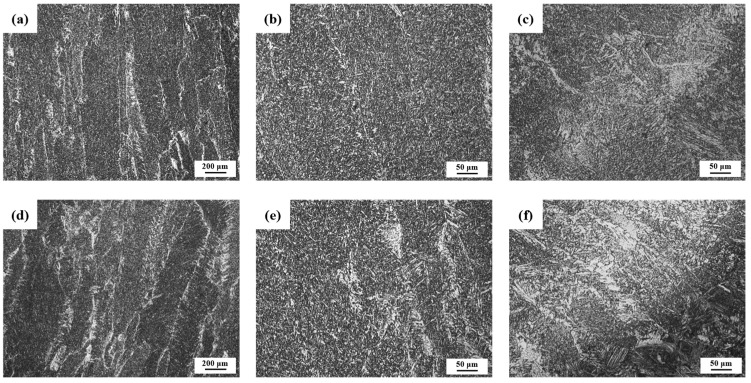
Microstructure of weld metal with different chromium content at different magnifications: (**a**) 1# Primary Solidification Zone 50×; (**b**) 1# Primary Solidification Zone 100×; (**c**) 1# Reheating Zone 100×; (**d**) 2# Primary Solidification Zone 50×; (**e**) 1# Primary Solidification Zone 100×; (**f**) 2# Reheating Zone 100×.

The primary solidification structure of the two-component weld was observed in the welding center area of the capping welding of the welded joints, as shown in Figure 3a,b,d,e. It can be seen that there is little difference in the dendrite size of the primary dendrite of the two-component welded joints, which is in the range of 200–300 µm. However, there are more white structures on the dendrite boundary of the 2# welded joint. Enlarging the white structure of the grain boundary, it can be clearly seen that the white chain from the grain boundary of the 1# weld is a small amount of proeutectoid ferrite, accounting for about 10%, and the grain size of the ferrite is relatively small, while the 2# dendrite boundary contains both white massive polygonal ferrite and the ferrite side plate growing in one side of the grain, and the volume fraction of the white structure accounts for more than 30%. At the same time, the reheat area at the junction of the capping welding and the previous weld pass was selected for comparative analysis of structure, as shown in Figure 3c,f. A large number of white structure areas appeared in the reheat zone of both welded joints, and the white area was mainly the ferrite side plate. But in general, the width of the white area in the 2# weld reheat zone is larger, the number of side lath ferrites in the white structure is more, and the ferrite grains are also larger.

The metallographic results show that the ferrite side plate of the 1# weld metal is smaller and finer than that of 2# weld metal. It can be seen that the 1# weld metal is mainly composed of acicular ferrite, while the 2# weld metal is mainly composed of the ferrite side plate and acicular ferrite, and the ferrite side plate accounts for more. With the addition of Cr element, the proportion of ferrite side plate in the 1# weld is reduced by about 20% compared with that in the 2# weld.

**Figure 4:**
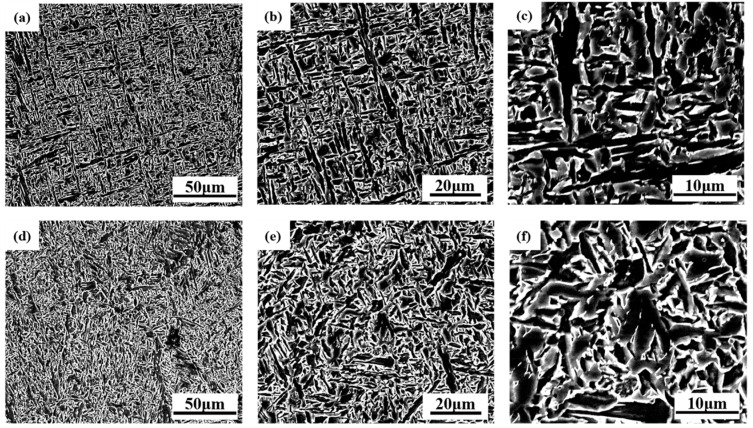
SEM microstructure characterization of two types of weld metal: (**a**) 1# 1000×; (**b**) 1# 2000×; (**c**) 1# 5000×; (**d**) 2# 1000×; (**e**) 2# 2000×; (**f**) 2# 5000×.

Figure 4 shows the microstructure of the two weld metals. Under the scanning electron microscope, the microstructure of the two weld metals shows acicular ferrite, but the size of the acicular ferrite in the 1# test weld metal is smaller and the “interlocking” form is more obvious. According to the preliminary analysis, this may be related to the content of Cr. Relevant studies have shown that with the increase in Cr content, the austenitic phase region of the test welding wire is reduced, the critical transition temperature is raised, and the formation of the ferrite phase is more favorable, resulting in the increase in acicular ferrite content in the final welded weld metal, and the overall microstructure is refined [32].

**Figure 5:**
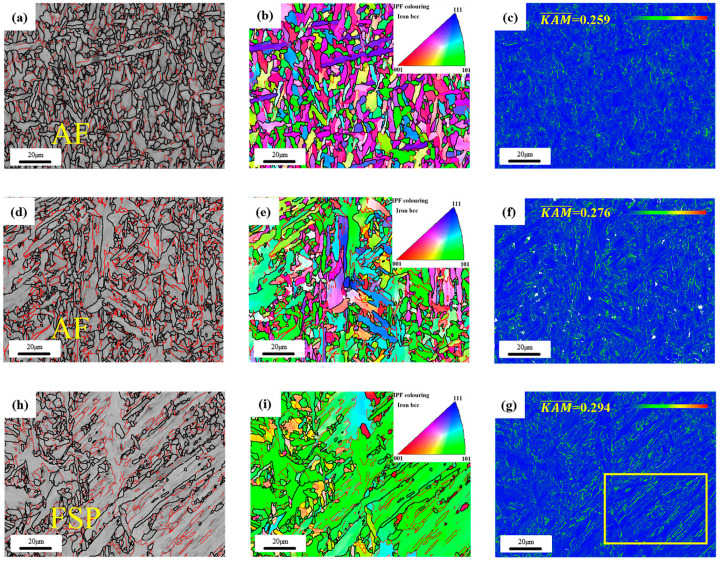
EBSD images of weld metal with different Cr contents: (**a**–**c**) acicular ferrite (AF) structure of 1# weld high- and low-angle grain boundary diagram; IPF diagram; KAM diagram; (**d**–**f**) acicular ferrite (AF) structure of 1# weld high- and low-angle grain boundary diagram; IPF diagram; KAM diagram; (**h**–**g**) ferrite side plate (FSP) structure of 2# weld high- and low-angle grain boundary diagram; IPF diagram; KAM diagram.

EBSD analysis was carried out on the two test wires. Figure 5 shows the high- and low-angle grain boundary diagram, inverse pole (IPF) diagram, and kernel average spread (KAM) diagram of the test wires. In the high- and low-angle grain boundary diagram, the red thin line represents the low-angle grain boundary with a difference of orientation between 2° and 15°, which is mainly the entanglement dislocation and some deformation defects in the structure. The thick black lines represent high angular grain boundaries with orientation differences greater than 15°, mainly representing the boundaries of the organization. The three orientations in the IPF diagram are related to the orientations of the structure, and the preferred orientations of the test wires can be seen through the IPF diagram. KAM diagram mainly reflects the stress concentration degree of welded joints surface. By comparing the high- and low-angle grain boundary diagrams of the two test welds, it can be found in Figure 5a,d,g that the high-angle grain boundary density of the 1# weld metal is significantly higher than that of the 2# weld metal, and the 1# weld metal presents fine grains, which are bounded by high-angle grain boundaries, while the 2# weld metal presents large grains, and some fine grains are distributed near the large grains. This also results in the high-angle grain boundary ratio and average grain size of the 2# weld metal being greater than that of the 1# weld metal. Figure 5b,e is the IPF diagram of the two weld metals. It can be seen that the acicular ferrite structure of the 1# weld metal is more uniform and finer than that of the 2# weld metal, and the orientation colors are separate from each other, which means that the adjacent acicular ferrite slats have different orientations, and the adjacent acicular ferrite grain orientations are different, form a relatively uniform “interlocking” acicular ferrite structure. Figure 5c,f,g is the KAM diagram. After comparison, it can be found that the stress concentration coefficient of the 1# weld metal is low, and the stress distribution is relatively uniform. The stress of the 2# weld metal is mainly concentrated at the boundary of the large slab structure, and the distribution similar to a “line” can be observed in the yellow box area of Figure 5h. When the welded joint is stressed, due to the stress difference between the acicular ferrite and the ferrite side plate grain boundary, the co-deformation ability of the weld metal will be weakened, and even become the crack source of intergranular fracture, resulting in the unsatisfactory toughness performance of the weld metal [33,34].

In Table 3, the proportions of large-angle grain boundaries and the transverse and longitudinal average grain sizes obtained from the analysis and statistics of the large-angle grain boundary maps of the two weld metals under different fields of view are obtained using Channel-5 software (5.12.74.00). The results show that the proportion of high-angle weld metal in weld 1# is 69%, and the proportion of large-angle weld metal in weld 2# is 59%. The average transverse grain size of weld metal is 2.01 μm, and the longitudinal effective grain size is 2.84 μm. All are lower than the average grain size of weld metal 2#. It can be seen that with the addition of Cr content, the proportion of high-angle grain boundary increases, and the effective grain size of the acicular ferrite structure of the test weld decreases by about 15%. Numerous studies have shown that high-angle grain boundaries in low-carbon micro-alloy steels can hinder crack propagation. When high-angle grain boundaries appear in the propagation path of microcracks, they often deviate or even completely prevent the propagation of microcracks, thereby improving the impact toughness of the test weld [35].

### 3.2. Mechanical Properties and Fracture Morphology of Weld Metal

**Figure 6:**
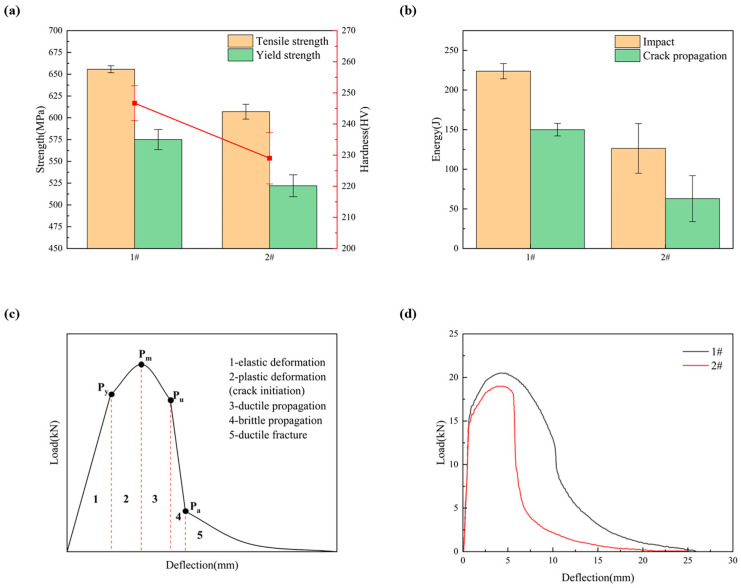
Different Cr content weld metal: (**a**) hardness and tensile properties; (**b**) impact energy; (**c**) complete load–deflection curve of ductile fracture; (**d**) experimental load–deflection curves of the 1# and 2# specimen.

The mechanical properties of the two weld metals are shown in Figure 6. Figure 6a shows the microhardness and tensile properties of the weld metal. In terms of hardness, the 1# weld metal is higher than the 2# weld metal. In terms of tensile properties, no matter the tensile strength (656 MPa) or yield strength (575 MPa) of the 1# weld metal is higher than that of the 2# weld metal, and the 1# weld metal has a yield ratio of 0.877, which is greater than that of the 2# weld metal.

In order to further analyze the toughness of weld metal samples, the instrument Charpy impact tests were carried out. Elastic deformation, plastic deformation (crack initiation), ductile growth (crack stable growth), brittle growth (crack unstable growth), and ductile fracture are the five stages of ductile fracture that are depicted in Figure 6c, the ideal impact load–deflection curve for ductile fracture [36,37]. Elastic deformation starts at the beginning of the impact test with a rather light load. Plastic deformation occurs at the notch root when the load is greater than the general yield load (Py). The start of the cross-section contraction takes place when the load reaches its maximum (Pm), at which point the plastic deformation extends throughout the impact specimen’s whole cross-section. This phase (stage 2) is when the crack starts to form. The fractures continue to spread when they are initiated until the load reaches Pu. Stage 3, also known as the stable propagation stage of the crack, is the phase in which the crack propagates with a significant energy expenditure. Since the fractures have already reached a critical size by the conclusion of Stage 3, unstable crack growth that is, the energy required for crack growth occurs in Stage 4. At last, ductile fracture occurs in stage 5, when the crack points reach the specimen’s compressive stress region while it is in the plane stress condition.

Figure 6d shows the weld metal specimens’ actual load–deflection curve. The impact toughness values and some typical values for the two specimens are listed in Table 4. The 1# weld metal exhibits a normal ductile fracture and is more consistent with the curve in Figure 6c. On the other hand, the 2# weld metal curve’s stage 3 (crack steady growth stage) is incredibly brief. The yield and peak load value of 2# weld metal are somewhat lower than those of 1# weld metal, and the load decrease is greater during the brittle fracture propagation stage. As shown in Table 4, the impact energy is consumed during crack initiation and crack propagation, where the sum of Ei and Ep values is almost equal to the impact toughness. The two weld metals exhibit distinct fracture propagation behaviors, though, and the Pa/Pm value is used to indicate the capacity to arrest cracks [38]. Specifically, a high Pa/Pm value indicates a high number of barriers that emerge along the propagation path. The 1# weld metal has a Pa/Pm value of 0.278, which is more than the 2# weld metal’s 0.216. These findings demonstrate that microcrack initiation for 2# weld metals causes rapid growth of crack instability because there are fewer barriers in the crack growth route, which causes a quick drop in material toughness [39].

Overall, the strength and toughness of 1# weld metal are better than that of 2# weld metal. The preliminary analysis shows that due to the difference in Cr content in 1# and 2# weld metal, with the addition of Cr element in 1# weld metal, the strength of test weld metal is improved, and the toughness of the weld joint is enhanced. For the test weld, the increase in weld metal strength is mainly due to the increase in the Cr element, which narrows the γ phase region, significantly changes the morphology distribution of ferrite, and refines the grain [32].

**Figure 7:**
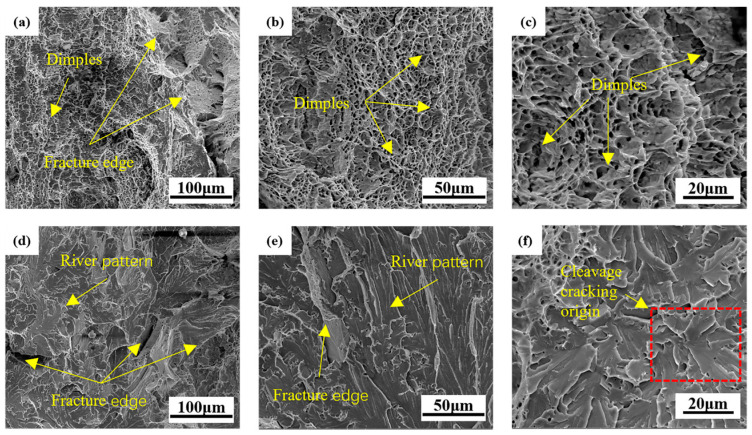
SEM Images of fracture surfaces of impact specimens at −20 °C: (**a**) 1# 500×; (**b**) 1# 1000×; (**c**) 1# 2000×; (**d**) 2# 500×; (**e**) 2# 1000×; (**f**) 2# 2000×.

Figure 7 shows the SEM photos of the impact fracture of the two welds. As can be seen from Figure 7, the 1# fracture morphology presents a typical dimple-like feature, which helps to prolong the phase of ductile crack growth (Figure 6b) and increase the energy absorbed by crack growth (Table 4), which is a characteristic manifestation of good toughness of the material [39]. The fracture morphology of the 2# weld metal is obviously river-like, and there are some torn dimples at the junction of the cleavage plane. In addition, there are some defects such as inclusions and holes on the cleavage surface, which is a typical brittle fracture of cleavage or quasi-cleavage.

## 4. Discussion

### 4.1. Effect of Cr Content on Microstructure of Weld Metal

**Figure 8:**
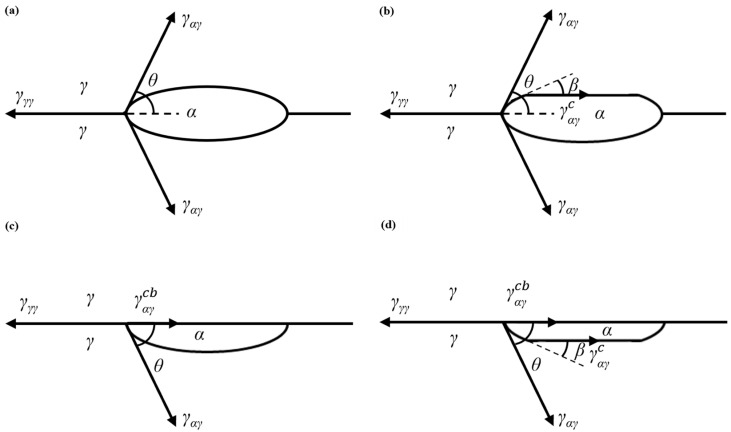
Nucleus models include spherical caps with disordered interphase boundaries of interfacial energy γ_αγ_; (**a**) two spherical caps that are abutting; (**b**) two spherical caps that are abutting, one of which is truncated by a low energy facet ${\;\gamma }_{\alpha \gamma }^{c}$; (**c**) one spherical cap that is accompanied by a facet with energy $\gamma_{\alpha \gamma }^{cb}$ that is low enough to lie in the grain boundary plane; (**d**) one spherical cap that is truncated by a facet of energy $\gamma_{\alpha \gamma }^{c}$ and is “capped” by a facet in the grain boundary plane.

The classical nucleation theory is applied to the solid–solid diffusion phase transition process, which is the most critical to determine the shape of critical nucleation [40]. Figure 8 shows a model of the spherical cap core forming a new solid phase at the former austenite grain boundary. Among them, Figure 8a is more consistent with the austenitic grain boundary ferritic nuclei of Fe-C alloys [26]. The nucleation free energy of austenite grain boundary is expressed in Formula (1) [41]:

(1) $$\Delta G=-V\Delta G_{V}+A_{\alpha \gamma }\gamma_{\alpha \gamma }\;-\;A_{\gamma \gamma }\gamma_{\gamma \gamma }$$

where $\Delta G_{V}$ is the volume free energy of the new phase α; V is the volume of the new phase; A_αγ_ is the interfacial area between the new phase α and the austenitic phase γ. γ_αγ_ is the interface energy between the new phase α and the austenitic phase γ. A_γγ_ is the interfacial area between the austenitic γ phases; γ_γγ_ is the interfacial energy between the austenitic γ phases.

According to Formula (1), when the content of Cr is small, it has little effect on the grain boundary energy ($A_{\gamma \gamma }\gamma_{\gamma \gamma }$), so the nucleation free energy is low, which is conducive to the grain boundary ferrite core, and the θ angle is reduced, less than 60° is conducive to the formation of grid structure of grain boundary ferrite structure along the austenite grain boundaries [41]. When the content of Cr in weld metal increases, the interfacial energy ($A_{\gamma \gamma }\gamma_{\gamma \gamma }$) decreases, resulting in the increase in nucleation free energy ΔG, which inhibits the nucleation of grain boundary ferrite. At the same time, the surface free energy ratio cosθ as shown in Formula (2) decreases [41], θ angle increases, the ferrite grows spherically or equiaxed on the austenite grain boundary, and the side lath ferrite decreases. This conclusion is consistent with the microstructure of weld metal observed in Figure 4. If the new phase formed is spherical, the interfacial free energy is significantly reduced, and θ in Figure 8a is:

(2) $$\cos\theta \;=\gamma_{\gamma \gamma }∕2\gamma_{\alpha \gamma }$$

**Figure 9:**
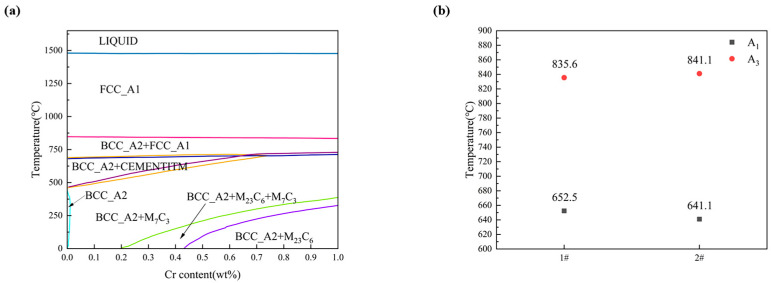
Thermodynamic properties diagram (**a**) Fe-Cr phase diagram (**b**) A1 and A3 temperatures at different.

The addition of Cr will also affect the phase transition behavior of weld metal. As shown in Figure 9, ignore slight fluctuations in element content in weld metal, the Thermo-Calc software (2023a) TCFE6 database is used to calculate Fe-Cr phase diagram at equilibrium at C = 0.05%, Mn = 1.3%, Si = 0.35%, Ti = 0.025, Ni = 0.8, Cu = 0.15 and Mo = 0.38 (mass fraction). When cooled to a low temperature, different types of carbides are formed during solid phase transition. As a strong carbide forming element, Cr will form carbides with carbon, and these fine dispersed carbides may be distributed on the austenite grain boundary, hindering the growth of austenite grains, resulting in small ferrite grain size in the final weld metal [32]. According to Table 3, it can also be seen that the effective grain size of ferrite in weld metals with high Cr content is small. In Figure 9b, Cr has little effect on the transition temperature of γ→α transition, but in the non-equilibrium state, Cr can hinder the diffusion of carbon atoms [42], delay the ferrite transition temperature, and inhibit the formation of side lath ferrite. No obvious ferrite side plate can be observed in Figure 4b.

### 4.2. Effect of Weld Metal Structure on Crack Propagation

#### 4.2.1. Critical Griffith Crack Size

The propagation of fracture instability only happens when the microcracks reach a crucial size. Crack sources in the weld can be identified by defects like hard inclusions and tiny voids. The occurrence of fracture instability growth is contingent upon the microcrack size resulting from these defects surpassing the critical crack size. As a result, the critical crack size must be determined. Formula (3) [43] can be used to calculate the dynamic yield stress value (δ_yd_) based on the general yield load P_y_ value.

(3) $$\sigma_{\mathrm{yd}}=467P_{y}∕B$$

where B is the Charpy impact specimen’s thickness. The dynamic yield stress in the impact test should be significantly higher than the static yield stress due to the strain rate effect. Therefore, the following Formula (4) [43] can be used to compute the critical cleavage stress (δ_c_):

(4) $$\sigma_{c}=c_{f}\sigma_{\mathrm{yd}}$$

Among these, 2.24 is considered to be the C_f_ value at the Charpy impact specimen’s notch [44]. The crucial Griffith microcrack size [36,37,38,39,44,45] can be calculated using Formula (5), which is based on Griffith theory:

(5) $$\sigma_{c}={\;\left(\frac{\pi E\gamma_{p}}{\left(1-v^{2}\right)d}\right)}^{1∕2}$$

E represents Young’s modulus, and γ_p_ denotes the microcrack’s effective surface energy, d is the critical crack length, and v is Poisson’s ratio. The values of E, γ_p_ and v are 210 GPa, 8–14 J/m^2^ and 0.3, respectively [36]. According to the variation range of γ_p_ value, the critical Griffith microcrack size of the 1# weld metal sample is about 2.3–4.0 μm, and the critical Griffith microcrack size of the 2# weld metal sample is about 2.6–4.5 μm. This seems to be contrary to the result of impact work, but the ratio of acicular ferrite in the 1# weld metal structure is larger, the grains are finer, and the density of high-angle grain boundaries is higher, which will prevent the crack propagation of brittle Griffith crack. When the high-angle grain boundary spacing is smaller than the critical Griffith crack size, it is difficult for the crack to expand to the critical size. As a result, the impact toughness is improved [46], which is also the reason why the toughness of the 1# weld metal structure does not deteriorate while the strength is increased.

#### 4.2.2. Crack Propagation Path

**Figure 10:**
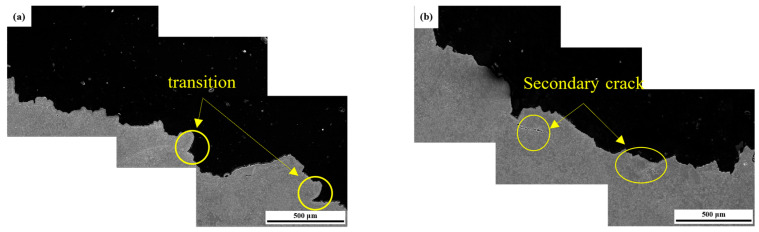

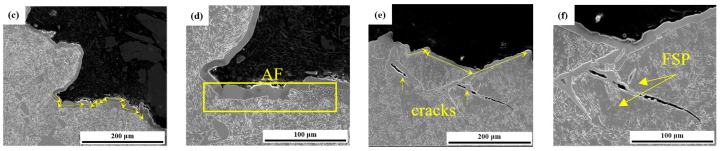
Fracture path of brittle zone in two types of weld metal impact fracture surfaces (**a**) 1# main crack; (**b**) 2# main crack; (**c**) 1# 500×; (**d**) 1# 1000×; (**e**) 2# secondary crack 500×; (**f**) 2# secondary crack 1000×.

The main crack trend in the brittle zone of the impact fracture of two kinds of weld metals is shown in Figure 10. Compared with Figure 10a,d, it can be found that the microstructure deformation of both is small. The microstructure of the 1# weld metal is mainly fine acicular ferrite, and no secondary cracks are generated in the main crack after several transitions, but more secondary cracks are generated in the 2# weld metal during the expansion of the main crack. Figure 10e,f are the enlarged images of the secondary cracks, and it is obvious that the cracks pass through large and complete grains, and almost no transitions occur. It ends in a small acicular ferrite structure.

**Figure 11:**
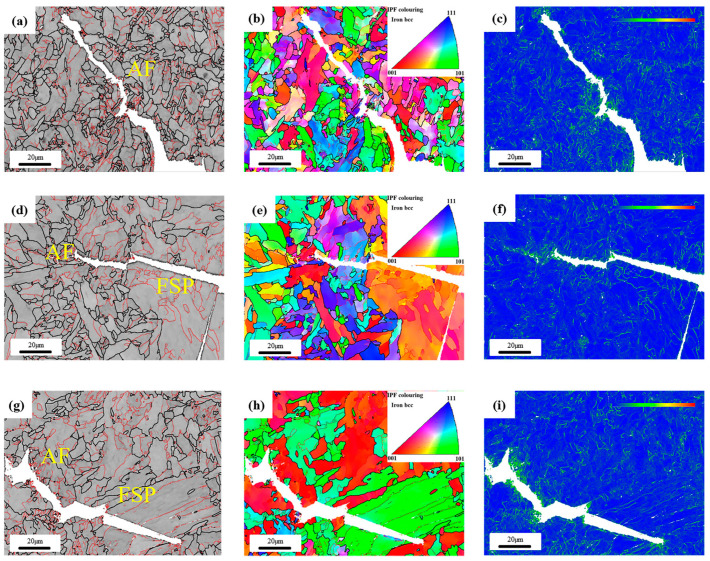
EBSD images of weld metal with different Cr contents: (**a**–**c**) 1# high- and low-angle grain boundary diagram; IPF diagram; KAM diagram; (**d**–**f**) 2# high- and low-angle grain boundary diagram; IPF diagram; KAM diagram. (**g**–**i**) 2# high- and low-angle grain boundary diagram; IPF diagram; KAM diagram.

Figure 11 shows the EBSD characterization of the crack growth path of weld metal after the instrument Charpy impact. As can be seen in Figure 11a, the 1# weld metal has a relatively high proportion of high-angle grain boundaries and a relatively small grain size, and the crack growth path is rather tortuous, which is manifested as deflection occurring at high-angle grain boundaries, and the crack growth consumes more energy [47,48,49,50]. Until the energy is exhausted, crack propagation stops. As shown in Figure 8b and Figure 11b,c, each of the 1# weld metal is uniformly oriented in reverse, forming an “interlocking” acicular ferrite, and the stress is also concentrated in the crack bend, that is, at the high-angle grain boundary, which also indicates that acicular ferrite effectively prevents crack expansion and improves the toughness of the weld metal.

As shown in Figure 11d,g, when the crack propagation meets the large side slat ferrite, it is almost not hindered, and the propagation path is very straight. During the process of passing through the grain, the energy required for crack propagation is very small, and the turning point does not occur until the high-angle grain boundary is encountered. In Figure 11e,h, it can be seen that the crack passes straight through a large piece of side lath ferrite with a single orientation. When there is a large amount of ferrite side plate in the weld metal, the weld has a poor ability to prevent crack propagation, which is also the reason for the poor toughness of the 2# weld metal.

## 5. Conclusions

Under the premise of low Cr content, this paper systematically studies the effect of whether Cr element is added to the gas-shielded solid wire on the microstructure and mechanical properties of the weld, and reaches the following conclusions:

1. Add 0.25 wt% Cr element in the welding wire, the tensile strength of the weld is increased from 607 MPa to 656 MPa, and the impact toughness is greatly increased from 126.37 J to 223.79 J. The optimum range of Cr content of gas-shielded solid wire is about 0.25%wt.
2. When a small amount (0.25%) of Cr is added to the weld, the proportion of ferrite side plate content in the weld structure is reduced by 20%, the acicular ferrite content is significantly increased, and the acicular ferrite grain size in the weld structure is reduced by about 15%.
3. With the increase in Cr content, the crack expansion of the weld consumes more energy, which is because the crack will be deflected when it meets the grain boundary at a high angle. The appropriate Cr element will increase the proportion of acicular ferrite, thereby increasing the density of the grain boundary at a high angle, preventing the crack from expanding, making its path more torsional, and ultimately leading to the strength and toughness of the weld.

## Figures and Tables

**Table 1:** Chemical compositions of welding wires.

No.	C	Si	Ti	Cr	Mn	Ni	Cu	Mo	Fe
Y1	0.047	0.43	0.026	0.25	1.35	0.81	0.15	0.38	Bal
Y2	0.041	0.42	0.025	/	1.30	0.84	0.14	0.38	Bal

**Table 2:** Chemical composition of weld cladding metal.

No.	C	Si	Ti	Cr	Mn	Ni	Cu	Mo	Fe
1#	0.052	0.39	0.016	0.25	1.26	0.78	0.14	0.36	Bal
2#	0.052	0.34	0.012	/	1.16	0.80	0.13	0.35	Bal

**Table 3:** Proportion of high-angle grain boundaries (HAGB) and effective grain size in transverse and longitudinal directions of weld metal.

No.	High-Angle Grain Boundary (%)	Transverse Effective Grain Size (µm)	Longitudinal Effective Grain Size (µm)
1#	65	2.01	2.84
2#	59	2.35	2.96

**Table 4:** Results of the instrumented Charpy impact test of the 1# and 2#.

Specimen	P_y_ (kN)	P_m_ (kN)	P_a_ (kN)	E_i_ (J)	E_p_ (J)	Impact Toughness (J)
1#	15.17	20.56	5.73	76.09	141.51	223.79 ± 9.57
2#	14.41	19.01	4.11	61.75	61.23	126.37 ± 31.38

P_y_: general yield load, P_m_: peak load, P_a_: fracture arrested load, E_i_: energy of crack initiation, E_p_: energy of crack propagation, impact toughness is calculated as the average of three specimens’ total absorbed energy.

## Data Availability

The original contributions presented in the study are included in the article, further inquiries can be directed to the corresponding author.

## References

[1] Liu Y., Liu J.F., Zhang B., Wang L.L., Sun S.Y., Hou J. (2024). Development status and trend of pipeline steel for long-distance natural gas transportation in China. Trans. Mater. Heat Treat..

[2] Gao Z.Y., Zhang H.Y., Gao P. (2023). New progress in China’s oil and gas pipeline construction in 2020. Int. Petroleum Econ..

[3] Feng Y.R., Huo C.Y., Ji L.K., Li H.L. (2016). Progress and prospects of research and applications of high grade pipeline steels & steel pipes in China. Pet. Sci. Bull..

[4] Zhao M., Li Q., Song H.Q. (2022). Research status and development trend of pipeline steel pipe welding technology. MW Met. Form..

[5] Sun P.X., Dai G.W., Li X.D. (2023). Application Status and Discussion of ECA Technology in Girth Welds of Oil and Gas Pipelines. Electr. Weld. Mach..

[6] Wang X.X. (2020). New Challenges of Pipeline Safety to Welded Pipe Manufacturing Technology. Welded Pipe Tube.

[7] Chen X.W., Zhang D.H., Wang X. (2021). New Challenges of Pipeline Safety to Welded Pipe Manufacturing Technology. Oil Gas Storage Transp..

[8] Dai L.S., Kao Q.P., Yang H. (2020). Hazard Control Measurement of Girth Weld in High Strength Steel Pipeline. Pet. Tubul. Goods Instrum..

[9] Wang M., Zhou C.L., Cao W. (2024). Current Status and Development Trends of Automation Technology for Long Distance Pipeline. Total Corros. Control.

[10] Quan T. (2021). Application Status and Prospect of Automatic Welding Technology for Long Distance Oil and Gas Pipelines in China. Chem. Enterp. Manag..

[11] Dong W.L., Pu J., Liu Z.M. (2022). Research on Welding Technique in Long-distance Pipeline Construction. China Spec. Equip. Saf..

[12] Wang J.Q., Qin H.B., Ye W. (2021). Automatic Welding Technology and Construction Application of Long-distance Oil and Gas Pipeline. Mod. Chem. Res..

[13] Lan J.X., Ma Q., Xiao H.Y. (2024). Research on the Welding Process of 1000 MPa Grade High Strength Steel Self-developed Solid Wire. Electr. Weld. Mach..

[14] Pei Z.Z., Chen X.H., Wang X.W. (2021). Microstructure and Mechanical Properties of ER70-Ti Steel for Gas Shielded Welding Wire. Mater. Electr. Weld. Mach. Sci. Forum.

[15] Hou Y. (2023). Effect of alloying element on microstructure and properties of girth welded joints of X80 pipeline steel. Metall. Anal..

[16] Qi L.H., Hu Y., Zhang S.J., Yang Y.B., Chen Y.F. (2023). Effect of Mo and Ni on Microstructure Properties and Deformation of X70 Pipe FCAW-G Girth Weld. Welded Pipe Tube.

[17] Cai J., Yang H.C., Zhang Y.F., Jia B. (2023). Effect of B element on the impact property of post-welding heat treatment of deposited metal. Weld. Cut..

[18] Chen R., Wang K., Yi Y.Y., Du X.D. (2017). Effects of RE Elements in Flux-cored Wire on Microstructure and Mechanical Properties of 921A Steel Weld Metal. Hot Work. Technol..

[19] Lu W.Z. (2021). Dynamic In-Situ Study on Microstructure Transformation in Welding Heat Affected Zone of Low Alloy High Strength Steel. Master’s Thesis.

[20] Hao Y.P., Li L., Gao Z.K., Ding W., Tan J.H., Liu W. (2022). Progress on the Welding of High-performance Bridge Steel. Adv. Mater. High Speed Railw..

[21] Xiao H.J., Tian Z.L., Cui B., Jiang Z.J. (2017). Effect of post weld heat treatment on microstructure and impact property of weld metal for Q460 steel. Heat Treat. Met..

[22] A-rong, Zhao L., Pan C., Tian Z.L. (2015). Influence of Ti on Weld Microstructure and Mechanical Properties in Large Heat Input Welding of High Strength Low Alloy Steels. Iron Steel Res. Int..

[23] Liu Z.J., Wu D., Su Y.H. (2018). Effect of boron element on formation of acicular ferrite in weld metal with flux cored wire. Trans. China Weld. Inst..

[24] Song M.M., Song B., Zhang S.H., Xue Z.L., Yang Z.B., Xu R.S. (2017). Role of Lanthanum Addition on Acicular Ferrite Transformation in C–Mn Steel. ISIJ Int..

[25] Kong H.Y., Zhu G.P., Zeng Z.W., Chun C., Yao R.G. (2017). Influence of Mo in Flux Cored Wires on the Mechanical Properties of Low Alloy Steel Weld Metal. Dev. Appl. Mater..

[26] Lee S.G., Lee D.H., Sohn S.S., Kim W.G., Um K.K., Kim K.S., Lee S. (2017). Effects of Ni and Mn addition on critical crack tip opening displacement (CTOD) of weld-simulated heat-affected zones of three high-strength low-alloy (HSLA) steels. Mater. Sci. Eng. A.

[27] Lee H.J., Lee H.W. (2015). Effect of Cr Content on Microstructure and Mechanical Properties of Low Carbon Steel Welds. Int. J. Electrochem. Sci..

[28] Wang F., Fan Y.R., Zhang X.X. (2014). Effect of Cr in Self-shielded Flux-cored Wires on Sharpy Impact Performance and Microstructure of the Weld Metals. Han Guan.

[29] Cui L., Gao Y., Gu C.S. (2018). Effect of Trace Element Cr on Microstructures and Properties of Welded Joints of Marine Corrosion Resisting Steels. J. Beijing Univ. Technol..

[30] Cai Y.C., Luo Z. (2015). Effect of Heat Chrome Element on Microstructure and Mechanical Properties of High-strength Steel Electrode Weld. Han Guan.

[31] (2019). Metallic Materials—Charpy V-Notch Pendulum Impact Test—Instrumented Test Method.

[32] Li H.C., Chen S.Y., Yue X.D., Yang H.M. (2014). Effect of Cr on microstructure and austenite-ferrite transformation temperature of low carton steel. Heat Treat. Met..

[33] Heo N.H., Heo Y.U., Kwon S.K., Kim N.J., Kim S.J., Lee H.C. (2018). Extended Hall–Petch Relationships for Yield, Cleavage and Intergranular Fracture Strengths of bcc Steel and Its Deformation and Fracture Behaviors. Met. Mater. Int..

[34] Wang T. (2016). Laser Welding Performance of Advanced Automotive High Strength Steels. Ph.D. Thesis.

[35] Huang L., Deng X.T., Liu J., Wang Z.D. (2017). Relationship Between Retained Austenite Stability and Cryogenic Impact Toughness in 0.12C-3.0Mn Low Carbon Medium Manganese Steel. Acta Metall. Sinca.

[36] Lan L.Y., Qiu C.L., Zhao D.W., Gao X.H., Du L.X. (2011). Microstructural characteristics and toughness of the simulated coarse grained heat affected zone of high strength low carbon bainitic steel. Mater. Sci. Eng. A.

[37] Luo X., Xu G., Chen X.H., Wang Z.D. (2022). Effect of undercooled austenite ausforming on the role of the M–A constituents in the CGHAZ toughness of the HSLA steels with bainite structure. Mater. Sci. Eng. A.

[38] Zhou Y.L., Jia T., Zhang X.J., Liu Z.Y., Misra R.D.K. (2015). Microstructure and toughness of the CGHAZ of an offshore platform steel. J. Mater. Process. Technol..

[39] Lan L.Y., Qiu C.L., Zhao D.W., Gao X.H., Du L.X. (2012). Analysis of microstructural variation and mechanical behaviors in submerged arc welded joint of high strength low carbon bainitic steel. Mater. Sci. Eng. A.

[40] Lange W.F., Enomoto M., Aaronson H.I. (1988). The kinetics of ferrite nucleation at austenite grain boundaries in Fe-C alloys. Metall. Trans. A.

[41] Porter D.A., Easterling K.E. (1973). Phase Transformations In Metals and Alloys.

[42] Zhu G.P., Guo C., Kong H.Y. (2018). Influence of Cr content in flux cored wire on mechanical properties of low alloy steel weld metal. Heat Treat. Met..

[43] Sreenivasan P.R., Ray S.K., Mannan S.L., Rodriguez P. (1996). Dynamic fracture toughness and Charpy impact properties of an AISI 403 martensitic stainless steel. J. Nucl. Mater..

[44] Schino A.D., Guarnaschelli C. (2009). Effect of microstructure on cleavage resistance of high-strength quenched and tempered steels. Mater. Lett..

[45] Li X.D., Ma X.P., Subramanian S.V., Shang C.J., Misra R.D.K. (2014). Influence of prior austenite grain size on martensite–austenite constituent and toughness in the heat affected zone of 700 MPa high strength linepipe steel. Mater. Sci. Eng. A.

[46] Agarwal P., Ramana V.G., Mishra D., Chandra A., Rathore G.S. DWTT in Thicker Gauge Hot Rolled Coils for Line Pipe Application. Proceedings of the 10th China Steel Annual Conference and the 6th Baosteel Academic Annual Conference.

[47] Hu J.T., Liu Y., Wang G., Li Q. (2021). Effects of Microstructure on the Low-Temperature Toughness of an X80 × D1422 mm Heavy-Wall Heat-Induced Seamless Bend. Met.—Open Access Metall. J..

[48] Zhou X.G., Li H., Chen Q.Y., Liu Z.Y. (2022). Controlled Rolling of X80 Pipeline Steel in the Austenite Recrystallization Temperature Region and Its Effect on the Microstructure and Mechanical properties. Steel Res. Int..

[49] Wu H.Y., Xia D.L., Du Y., Gao G.R., Gao X.H., Du L.X., Ma H. (2022). Study on Microstructure Characterization and Impact Toughness in the Reheated Coarse-Grained Heat Affected Zone of V-N Microalloyed Steel. J. Mater. Eng. Perform..

[50] Xu K., Fang T., Zhao L.F., Cui H.C., Liu F.G. (2020). Effect of Trace Element on Microstructure and Fracture Toughness of Weld Metal. Acta Metall. Sinca-Engl. Lett..

